# Protein kinase B controls *Mycobacterium tuberculosis* growth via phosphorylation of the transcriptional regulator Lsr2 at threonine 112

**DOI:** 10.1111/mmi.14398

**Published:** 2019-10-10

**Authors:** Kawther Alqaseer, Obolbek Turapov, Philippe Barthe, Heena Jagatia, Angélique De Visch, Christian Roumestand, Malgorzata Wegrzyn, Iona L. Bartek, Martin I. Voskuil, Helen M. O'Hare, Paul Ajuh, Andrew R. Bottrill, Adam A. Witney, Martin Cohen‐Gonsaud, Simon J. Waddell, Galina V. Mukamolova

**Affiliations:** ^1^ Leicester Tuberculosis Research Group Department of Respiratory Sciences University of Leicester Leicester LE2 9HN UK; ^2^ Department of Basic Science Faculty of Nursing University of Kufa Najaf Governorate P.O. Box 21 Kufa Najaf Iraq; ^3^ Centre de Biochimie Structurale CNRS INSERM University of Montpellier 34090 Montpellier France; ^4^ Wellcome Trust Brighton and Sussex Centre for Global Health Research Brighton and Sussex Medical School University of Sussex Brighton BN1 9PX UK; ^5^ Core Biotechnology Services University of Leicester University Road Leicester LE1 7RH UK; ^6^ Department of Immunology and Microbiology University of Colorado School of Medicine Aurora CO 80045 USA; ^7^ LISCB Department of Molecular and Cell Biology University of Leicester University Road Leicester LE1 7RH UK; ^8^ Gemini Biosciences Ltd Liverpool Science Park Liverpool L7 8TX UK; ^9^ Protein Nucleic Acid Laboratory University of Leicester Leicester LE1 7RH UK; ^10^ Institute for Infection and Immunity St George's University of London London SW17 0RE UK

## Abstract

*Mycobacterium tuberculosis* (*Mtb)* is able to persist in the body through months of multi‐drug therapy. Mycobacteria possess a wide range of regulatory proteins, including the protein kinase B (PknB) which controls peptidoglycan biosynthesis during growth. Here, we observed that depletion of PknB resulted in specific transcriptional changes that are likely caused by reduced phosphorylation of the H‐NS‐like regulator Lsr2 at threonine 112. The activity of PknB towards this phosphosite was confirmed with purified proteins, and this site was required for adaptation of *Mtb* to hypoxic conditions, and growth on solid media. Like H‐NS, Lsr2 binds DNA in sequence‐dependent and non‐specific modes. PknB phosphorylation of Lsr2 reduced DNA binding, measured by fluorescence anisotropy and electrophoretic mobility shift assays, and our NMR structure of phosphomimetic T112D Lsr2 suggests that this may be due to increased dynamics of the DNA‐binding domain. Conversely, the phosphoablative T112A Lsr2 had increased binding to certain DNA sites in ChIP‐sequencing, and *Mtb* containing this variant showed transcriptional changes that correspond with the change in DNA binding. In summary, PknB controls *Mtb* growth and adaptations to the changing host environment by phosphorylating the global transcriptional regulator Lsr2.

## Graphical abstract

Protein kinase B (PknB) is essential for growth of *Mycobacterium tuberculosis* because of its central role in regulation of peptidoglycan biosynthesis. Here we describe that PknB, controls activation of alternative pathways that are not required for active growth but crucial for survival in stressful environment and virulence. PknB phosphorylates Lsr2, a global transcriptional regulator, at threonine 112 and controls its binding to DNA and transcription of target genes.

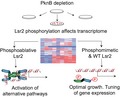

## Introduction

*Mycobacterium tuberculosis* (*Mtb*) is a slow‐growing bacterium that can replicate in humans and cause tuberculosis. The pathogen is able to rapidly shut down its growth to persist in non‐replicating states in infected individuals, which can be modelled in the laboratory (Wayne and Sohaskey, 2001). *Mtb* adaptation to non‐permissive conditions is accompanied by dramatic changes in global protein phosphorylation but the importance of these modifications is poorly defined (Prisic *et al.*, 2010). *Mtb* has 11 serine/threonine protein kinases and they play significant roles in growth, virulence and metabolism (Richard‐Greenblatt and Av‐Gay, 2017). In particular, protein kinase B (PknB) is reported to be essential for *Mtb* growth (Fernandez *et al.*, 2006; Forti *et al.*, 2009) due to its critical function in the regulation of peptidoglycan biosynthesis (Gee *et al.*, 2012; Boutte *et al.*, 2016; Turapov *et al.*, 2018). It is also important for *Mtb* survival in hypoxic conditions and resuscitation during reaeration (Ortega *et al.*, 2014). However, the molecular mechanism for PknB‐mediated adaptation to hypoxia is unknown.

We have recently shown that PknB‐depleted *Mtb* can grow in osmoprotective sucrose magnesium medium (SMM) (Turapov *et al.*, 2018). Comparative phosphoproteomic analysis of PknB‐producing against PknB‐depleted mycobacteria revealed substantial changes. Specifically, the transcriptional regulator Lsr2 showed increased phosphorylation in PknB‐producing mycobacteria, indicating that this protein may be a PknB substrate.

Lsr2 is a DNA‐binding protein that combines the properties of a nucleoid‐associated protein (Kriel *et al.*, 2018) and a global transcriptional regulator (Bartek *et al.*, 2014). Lsr2 has over 1000 binding sites in *Mtb* (Gordon *et al.*, 2010; Minch *et al.*, 2015). The precise role of Lsr2 in mycobacterial biology remains unclear, nevertheless parallels may be drawn with similar proteins from other bacteria. Lsr2 represents the first example of an H‐NS‐like protein identified outside Gram‐negative bacteria; moreover, *lsr2* was able to complement an *hns* mutant in *Escherichia coli* (Gordon *et al.*, 2008). Similar to the H‐NS proteins, Lsr2 has been proposed to bind to the minor groove of DNA (Gordon *et al.*, 2011) and to possess DNA bridging properties (Chen *et al.*, 2008). Additionally, Lsr2 has been shown to protect DNA from reactive oxygen species, and overexpression of Lsr2 improved survival of mycobacteria treated with hydrogen peroxide (Colangeli *et al.*, 2009). Deletion of *lsr2* in *Mtb* resulted in severe growth impairment on solid media, defects in persistence and adaptation to changing oxygen levels, all of which were accompanied by differential expression of genes involved in cell wall remodelling, respiration and lipid biosynthesis (Bartek *et al.*, 2014).

Here, we profiled the transcriptional changes that resulted from PknB depletion, and investigated the role of Lsr2 in coordinating these changes, as suggested by reduced phosphorylation of Lsr2 at T112 during PknB depletion (Turapov *et al.*, 2018). We probed the role of phosphorylation at this site in regulation of the structure and DNA‐binding properties of Lsr2 and in governing growth and survival of *Mtb* in different conditions. Based on our data, we propose that PknB‐mediated phosphorylation controls Lsr2 binding to DNA in *Mtb*, providing a functional link between serine/threonine protein kinase signalling in replicating bacilli and regulatory networks that enable *Mtb* to survive dynamic environments during infection.

## Results

### Transcriptome profiling of PknB‐depleted *Mtb* revealed an Lsr2‐regulated gene expression signature

**Figure 1:**
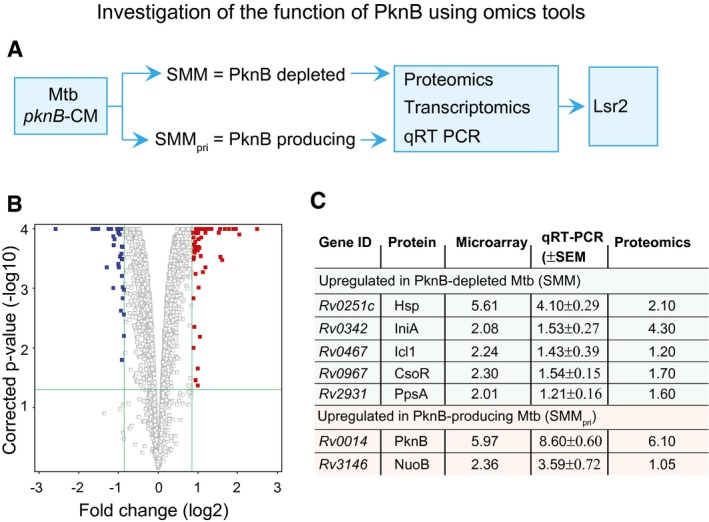
Application of omics tools to characterise the function of *pknB* essential in standard growth media without affecting *Mtb* viability. A. Experimental set‐up for sample preparation and analysis. Conditional PknB mutant (*pknB*‐CM) was grown in sucrose‐magnesium medium with pristinamycin (SMMpri, activation of PknB expression) or without pristinamycin (SMM, PknB depletion). Phosphoproteomics analysis was previously described (Turapov et al., 2018). B. PknB depletion results in alteration of *Mtb* transcriptome. Transcriptional impact of PknB depletion in *Mtb*. Volcano plot showing 65 genes significantly induced (red) and 34 genes repressed (blue) by PknB‐depletion in replicating *Mtb*. Significantly differentially expressed genes were identified using a moderated *t*‐test (*P*‐value < 0.05 with Benjamini and Hochberg multiple testing correction) and fold change >1.8 from three biological replicates. Fold change (log2) comparing *Mtb pknB*‐CM in SMM with and without pristinamycin is plotted on the *x*‐axis; corrected *p*‐value (−log10) on the *y*‐axis. Application of TFOE tool Lsr2 as potential regulator of the observed gene expression patterns Lsr2. C. Expression of selected targets was validated using qRT‐PCR and proteomics approaches (see for detail Experimental procedures). Expression of *pknB*, *nuoB*, *hsp*, *csoR* was significantly different in SMM compared with SMMpri (*P* < 0.01, *t*‐test).

PknB is essential for growth in standard conditions; however, we have recently developed an osmoprotective medium (SMM) that supported growth of PknB‐depleted *Mtb* and allowed us to identify PknB substrates (Turapov *et al.*, 2018). Using the same system, we compared the transcriptional profile of PknB‐depleted versus PknB‐producing *Mtb* (Fig. 1A, Tables 1 and S1). PknB‐depletion led to specific and significant changes in gene expression: 65 genes were induced and 34 repressed (Fig. 1B, Table S1). Two functional classes were overrepresented amongst the induced genes compared to the genome as a whole: regulatory proteins and proteins involved in lipid metabolism. The induced genes annotated as transcriptional regulators were *csoR*, *rv1129c*, *rv1460*, *rv2017*, *rv2250c*, *rv3334*, *sigB*, *whiB3* and *whiB6* (Table S1). These transcription factors regulate copper homoeostasis (CsoR) (Marcus *et al.*, 2016), iron–sulphur cluster biogenesis (Rv1460) (Willemse *et al.*, 2018), cholesterol catabolism (Rv1129c/PrpR) (Masiewicz *et al.*, 2012), the enduring hypoxic response (Rv3334) (Rustad *et al.*, 2008), multiple stress responses (SigB) (Lee *et al.*, 2008), redox stress and complex lipid biosynthesis (WhiB3) (Mehta and Singh, 2018) and virulence factor expression (WhiB6) (Bosserman *et al.*, 2017).

**Table 1:** The impact of PknB depletion on the gene and protein expression levels of serine/threonine protein kinases and Lsr2 in *Mtb*.

Gene	Protein	Description	Transcriptomics	Proteomics
			Fold difference SMMpri vs SMM^a^	Fold difference SMMpri vs SMM
*Rv3597c*	Lsr2	Protein Lsr2	1.01	1.20
*Rv0015c*	PknA	Protein kinase PknA	1.27	0.80
*Rv0014c*	PknB	Protein kinase PknB	5.97	6.60
*Rv0931c*	PknD	Protein kinase PknD	1.02	0.70
*Rv1743*	PknE	Protein kinase PknE	1.04	1.00
*Rv1746*	PknF	Protein kinase PknF	0.77	0.40
*Rv0410c*	PknG	Protein kinase PknG	0.93	1.30
*Rv1266c*	PknH	Protein kinase PknH	1.02	0.70
*Rv2914c*	PknI	Protein kinase PknI	0.84	N/D
Rv2088	PknJ	Protein kinase PknJ	0.89	N/D
Rv3080c	PknK	Protein kinase PknK	1.07	N/D
Rv2176	PknL	Protein kinase PknL	0.90	N/D

a Fold change values, comparing *Mtb* *pknB*‐CM grown in sucrose magnesium medium with (SMMpri) or without pristinamycin (SMM) are derived from microarray and proteomics profiles.

The transcriptional signature of PknB depletion resembled features of intracellular growth (Table S1), with a significant overlap with RNA profiles from several studies of *Mtb* in macrophages as reflected by hypergeometric probability values: 6.7 × 10^−23^ (Tailleux *et al.*, 2008), 7.34 × 10^−18^ (Schnappinger *et al.*, 2003) and 3.57 × 10^−17^ (Rohde *et al.*, 2007). For example, there was induction of pathways involved in mycobactin synthesis (*mbtB*/*C*/*D*), complex lipid phthiocerol dimycocerosate (PDIM) biosynthesis (*fadD26*, *ppsA*/*B*/*C*/*D*), metabolism of alternative lipid carbon sources, the glyoxylate shunt (*icl*), the methylcitrate cycle (*prpD*/*C*, *prpR*) and triacylglycerol synthase (*tgs1*). The isoniazid inducible genes (*iniB*/*A*/*C*) that respond to cell wall stress (Colangeli *et al.*, 2007), and four of the nine genes coding for alternative ribosomal proteins, *rpmB1*, *rpmB2*, *rpmG1*, *rpsN2* (Prisic *et al.*, 2015) were also induced.

The 34 genes that were significantly repressed in PknB‐depleted bacteria included *pknB* itself (sixfold change, whereas no other protein kinases were significantly changed Table 1); *nuoA/B/C*, encoding subunits of NADH dehydrogenase I, which is part of the aerobic respiratory chain, and several genes involved in intermediary metabolism (Table S1). Comparison of gene expression and protein abundance of selected targets showed good agreement (Fig. 1C). Overall the number of differentially expressed genes was comparable to the number with differential expression when other regulators were similarly disrupted, for example DosR (Park *et al.*, 2003). This is in contrast to the large‐scale changes in gene expression after treatment with an inhibitor of PknB and PknA (Carette *et al.*, 2018), which would likely impact *Mtb* viability. In summary, PknB depletion in replicating bacteria resulted in co‐ordinated changes to the transcriptome with similarities to intracellular adaptations, suggesting that PknB may control the induction of alternative gene regulatory pathways.

Application of the Transcription Factor Over‐Expression (TFOE) output tool (Rustad *et al.*, 2014) predicted Rv0081 (Galagan *et al.*, 2013), DosR (Park *et al.*, 2003) and Lsr2 (Bartek *et al.*, 2014) as potential regulators of the observed gene expression patterns (Fig. 1, Table S1). We next focussed on the involvement of Lsr2 in PknB‐mediated transcriptional adaptation, since Lsr2 was identified as a putative PknB substrate in our earlier phosphoproteomic work (Turapov *et al.*, 2018): PknB depletion decreased Lsr2 phosphorylation 2.54‐fold without impacting Lsr2 protein expression levels (Turapov *et al.*, 2018; Table 1). By contrast, the phosphorylation of DosR was unchanged during PknB depletion, and there are no reports of regulation of Rv0081 by phosphorylation.

### PknB phosphorylated Lsr2 *in vitro*

**Figure 2:**
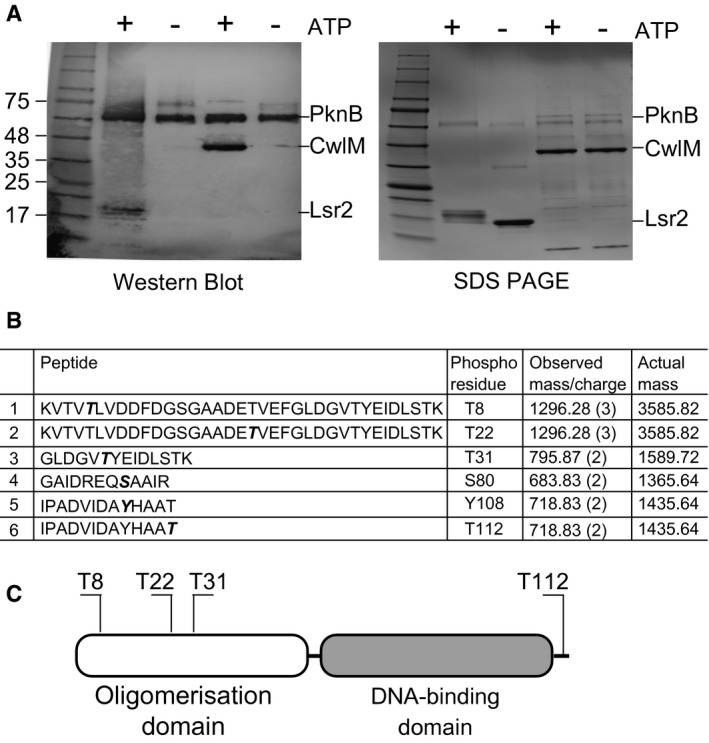
Identification of Lsr2 as a substrate of PknB. A. Recombinant Lsr2 was phosphorylated by recombinant PknB_KD_. Phosphorylated proteins were detected by western blot using a phospho‐threonine antibody. Recombinant CwlM was used as positive control. 1 – protein markers; 2 – Lsr2 incubated with PknB and ATP; 3 – Lsr2 incubated with PknB without ATP; 4 – CwlM incubated with PknB and ATP; 5 – CwlM incubated with PknB without ATP. B. SDS‐PAGE revealed a shift in Lsr2 mobility upon phosphorylation (lanes identical to panel A). C. Schematic presentation of phosphosites identified in phopshoproteomics studies (top) (Turapov et al., 2018), and *in vitro* (bottom). D. Phosphopeptides detected by mass spectrometry; phosphorylated residues shown in bold font.

Kinase assays of purified PknB kinase domain with Lsr2 used anti‐phosphothreonine antibody to detect phosphorylation and demonstrated that Lsr2 was directly phosphorylated by PknB (Fig. 2A). Interestingly, phosphorylation resulted in a marked change in Lsr2 protein mobility in SDS‐PAGE (Fig. 2B) and generated several bands, indicative of multiple phosphorylated forms. Mass spectrometry confirmed the previously observed phosphosite on threonine 112 (Turapov *et al.*, 2018) and detected additional phosphorylations at threonine 8, threonine 22 and threonine 31 (Fig. 2C and D).

### Phosphosite threonine 112 was necessary for Lsr2 function in *Mtb*

**Figure 3:**
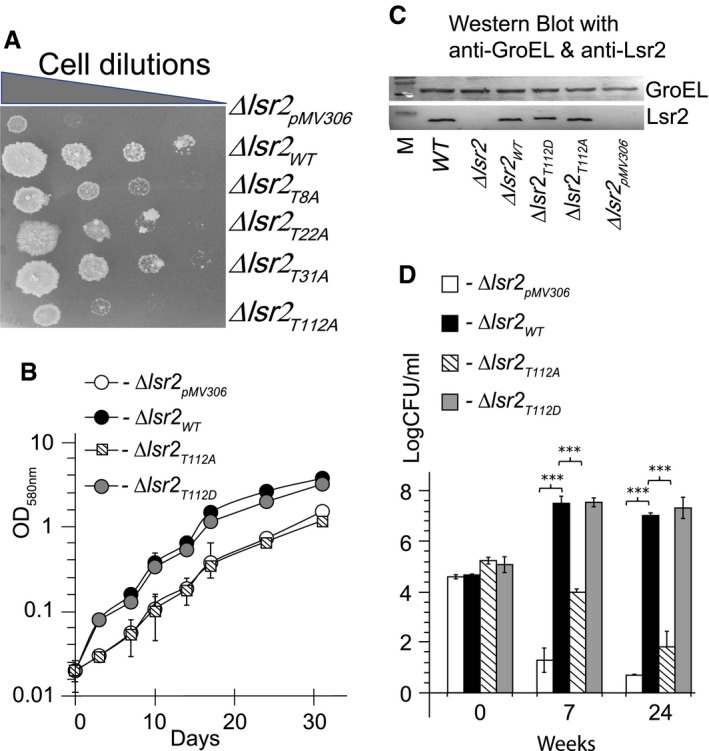
Phosphoablative T112A variant does not complement growth and survival defect of *lsr2* deletion mutant. A. Lsr2 phosphoablative mutants were serially diluted and plated on 7H10 agar. Growth of *lsr2* deletion mutant expressing wild‐type, T8A, T22A, T31A and T112A variants was compared with growth of the deletion mutant containing the empty vector on 7H10 agar. Experiment was repeated using two biological replicates. B. *Mtb* Δ*lsr2_pMV_*, Δ*lsr2_WT_*, Δ*lsr2_T112A_* and Δ*lsr2_T112D_* (~6 × 10^5^ cells/ml of each strain) were inoculated in 7H9 liquid medium supplemented with ADC and Tween 80 and incubated at 37ºC without shaking. C. Expression of Lsr2 and Lsr2 variants from pMV306 was verified by western blotting of *Mtb* lysates using an anti‐Lsr2 antibody. Expression of GroEL was used as loading control. D. T112A mutation impairs *Mtb* survival in the Wayne model of non‐replicating persistence. *Mtb*Lsr2 mutants were incubated in sealed tubes with gentle mixing for up to 24 weeks. B and D Data presented as mean ± SEM (*N* = 6, two independent experiments done with biological triplicates). ***Statistically different in Δ*lsr2_pMV_* or Δ*lsr2_T112A_* compared with Δ*lsr2_WT_* and Δ*lsr2_T112D_*_._

The functional importance of the identified phosphorylation sites in Lsr2 was further investigated by constructing a panel of phosphoablative *lsr2* variants using the pMV306 plasmid that integrates at *attB* site of *Mtb* chromosome (Table S2) and measuring their ability to complement the phenotypic changes caused by *lsr2* deletion in *Mtb. Lsr2* deletion mutant containing the empty pMV306 plasmid (Δ*lsr2*_pMV306_) was used as a control. *Lsr2* deletion significantly impaired *Mtb* growth on solid media, similarly to a previous study (Bartek *et al.*, 2014). Expression of wild‐type *lsr2* allele at *attB* site (the resultant strain designated as Δ*lsr2*_WT_) fully complemented the defect (Figs 3A and S1A). *Lsr2* deletion mutant expressing phosphoablative T112A Lsr2 variant (Δ*lsr2*_T112A_) had impaired growth on solid media (Figs 3A and S1A), whereas all other phosphoablative variants (T8A, T22A, T31A) complemented growth fully (Fig. 3A). Growth of *lsr2* deletion mutant expressing a T112D phosphomimetic variant of Lsr2 (Δ*lsr2*_T112D_) was indistinguishable from growth of wild‐type *Mtb* (Fig. S1A). Notably, Δ*lsr2*_pMV306_ and Δ*lsr2*_T112A_ transformants were recovered from liquid medium, since these strains failed to produce colonies on solid media unlike transformation with plasmids carrying wild‐type *lsr2* and other variants in Fig. 3.

Comparison of the growth rates in liquid 7H9 medium (Fig. 3B) revealed a similar pattern: the phosphomimetic T112D variant grew at a similar rate to the strain with wild‐type Lsr2 (Δ*lsr2*_T112D_ 0.039 ± 0.009 h^−1^ and Δ*lsr2*_WT_ 0.041 ± 0.004 h^−1^), whereas the phosphoablative T112A variant had a similar defect to the plasmid control (Δ*lsr2*_T112A_ 0.017 ± 0.004 h^−1^ and *Δlsr2*_pMV306_ 0.016 ± 0.03 h^−1^). This failure of Lsr2 T112A to complement the growth defect of Δ*lsr2* in liquid or solid medium, despite expression at similar levels (Fig. 3C), indicates a requirement for phosphorylatable threonine at this position for the function of Lsr2 in growing *Mtb*, suggesting a possible mechanism by which PknB might regulate transcription via phosphorylation of Lsr2. The phosphomimetic T112D Lsr2 had reduced mobility in SDS PAGE, similar to phosphorylated Lsr2 (Figs 3C and 2B).

In separate experiments, we investigated whether expression of T112D Lsr2 from pMV306 would complement the growth defect of *pknB*‐CM on solid SMM agar. However, introduction of pMV306::*lsr2*_WT_ or pMV306::*lsr2*_T112D_ did not improve growth of *pknB*‐CM; moreover pMV306::*lsr2*_T112A_ failed to produce any transformants, suggesting that T112A Lsr2 was toxic for *pknB*‐CM.

### Lsr2 phosphorylation at threonine 112 is crucial for *Mtb* adaptation to hypoxic conditions but not in prolonged stationary phase

We hypothesised that regulation of Lsr2 by PknB could account for the defects in survival of oxygen depletion reported when *pknB* and *lsr2* function was abrogated (Bartek *et al.*, 2014; Ortega *et al.*, 2014). Thus, we assessed survival of *Mtb* carrying Lsr2 variants using the Wayne model of non‐replicating persistence in hypoxia (Wayne and Sramek, 1994). The viable counts of Δ*lsr2*_WT_ and Δ*lsr2*_T112D_ increased after 7 weeks of incubation, proving that *Mtb* grew during gradual depletion of oxygen. In opposite, Δ*lsr2*_pMV306_ showed a dramatic drop of CFU counts below the initial inoculum. Further incubation for 24 weeks resulted in near‐complete loss of Δ*lsr2*_pMV306_ bacteria but did not significantly alter the survival of Δ*lsr2*_WT_ and Δ*lsr2*_T112D_. Interestingly, T112A Lsr2 was apparently unable to grow during initial 7 weeks, suggesting a requirement for T112 phosphorylation during adaptation for decreasing oxygen levels.

We also assessed the survival of *Mtb* expressing Lsr2 variants in late stationary phase of aerobically grown cultures by MPN and CFU counting. Δ*lsr2*_pMV306_ showed impaired survival compared to Δ*lsr2*_WT_, resulting in a 1.5 order of magnitude difference in viable counts. In this model, the survival of T112A or T112D variants (Δ*lsr2*_T112A_, Δ*lsr2*_T112D_) was not significantly different from wild‐type Lsr2 (Fig. S1B). Our results suggest that phosphorylation of Lsr2 at T112 may be specifically required during adaptation to hypoxic conditions but not for survival in prolonged stationary phase.

### Increased DNA binding in the T112A Lsr2 variant of *Mtb*‐altered gene expression

Phosphorylation of nucleoid‐associated proteins is known to influence their interaction with DNA (Dilweg and Dame, 2018), thus providing a mechanism by which PknB might regulate transcription via Lsr2. The DNA‐binding profile of Lsr2, and the influence of phosphorylation upon its DNA‐binding profile, were investigated using ChIP‐seq and a custom anti‐Lsr2 antibody. Lsr2‐binding peaks were found at intergenic spaces and running through several open reading frames, and were well conserved across biological replicates (Fig. S2 and Table S3), whereas no DNA was precipitated from strains lacking *lsr2*, confirming antibody specificity. The putative regulon of Lsr2, including genes with a Lsr2‐binding site within or immediately upstream of the coding sequence, was defined as 1178 genes (Table S3), and was consistent with previously identified Lsr2‐binding patterns (Gordon *et al.*, 2010; Minch *et al.*, 2015) (Table S4). The regulon was significantly enriched in genes that are differentially expressed upon inactivation of Lsr2 (Bartek *et al.*, 2014), macrophage infection (Tailleux *et al.*, 2008), in sputum (Garton *et al.*, 2008) and under acid‐nitrosative stress (Cossu *et al.*, 2013; Table S4), overlapping with the transcriptional signature of PknB depletion.

**Figure 4:**
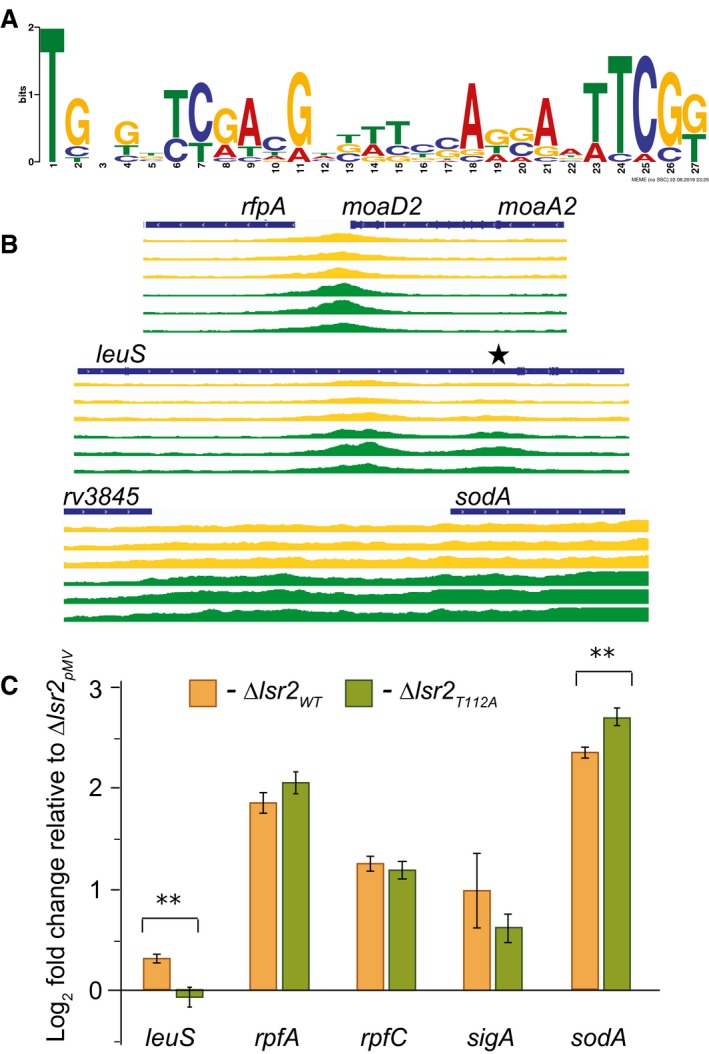
T112A mutation alters Lsr2 binding to DNA and gene expression patterns. A. Lsr2 T112A binding site was predicted using the MEME Suite (://meme-suite.org) and sequence of 25 DNA fragments which were >6‐fold enriched in Lsr2 T112A compared with Lsr2 WT. B. Representative plots describe Lsr2 binding upstream of *rpfA*, intragenic binding in *leuS*, or both intergenic and intragenic binding in *sodA*, showing greater Lsr2 binding in three biological replicates of phosphoablative Δ*lsr2_T112A_* (green) compared to Δ*lsr2_WT_* (yellow). A black asterisk marks the position of a Lsr2‐binding site in *leuS*. Plots adapted from Integrative Genomics Viewer IGV, (Robinson et al., 2011). Binding patterns in Δ*lsr2_T112D_* were identical to those in Δ*lsr2_WT_* and not shown for clarity. C. Expression of *leuS*, *rpfA*, *rpfC*, *sigA* and *sodA* relative to Δ*lsr2_pMV306_*measured by quantitative RT‐PCR in Δ*lsr2_WT_* and Δ*lsr2_T112A_* and normalised to *16s rRNA and* Δ*lsr2_pMV306_*. Data presented as mean ± SEM (*N* = 6). **Statistically different in Δ*lsr2_T112A_* compared with Δ*lsr2_WT_* (*P* < 0.01).

To measure the effect of phosphorylation of Lsr2 on its DNA‐binding pattern, we compared the ChIP‐seq data above with parallel experiments using phosphomimetic Lsr2 (Δ*lsr2*_T112D_) and phosphoablative Lsr2 (Δ*lsr2*_T112A_). The results for phosphomimetic strain were essentially the same as those with wild‐type Lsr2 (Δ*lsr2*_WT_ *Mtb*, no significant differences in sequence abundance), whereas DNA precipitated from the phosphoablative strain showed a significant increase in the abundance of 226 putative Lsr2‐binding sites (Fig. S2), suggesting that greater DNA binding of this variant. These binding sites may affect the expression of 94 genes (Table S5), which are involved in pathways of cell wall biosynthesis, lipid metabolism, PE/PPE protein synthesis and intermediary metabolism. Application of Motif‐based sequence analysis predicted a binding site where T122A variant was preferably binding (Fig. 4A).

To determine whether altered binding of Lsr2 variants resulted in corresponding changes in RNA abundance, we tested two of these genes for differential expression in the presence/absence of Lsr2 and in the presence of wild‐type versus phosphoablative Lsr2. *sodA* and *leuS* were selected for investigation on the basis of their divergent patterns of Lsr2 binding (Fig. 4B). *sodA* was upregulated but *leuS* did not change significantly in Δ*lsr2*_WT_ compared with Δ*lsr2_pMV306_* (Fig. 4C). Both genes were differentially expressed in Δ*lsr2_T112A_* versus Δ*lsr2*_WT_, but *leuS* expression was lower, while *sodA* expression was higher (Fig. 4C). These results suggest that the influence of phosphorylation on Lsr2 DNA binding can up‐ or downregulate gene expression, depending on promoter structure and other regulatory factors. Genes known to be differentially expressed upon *lsr2* disruption were included alongside (*rpfA* and *rpfC*) (Bartek *et al.*, 2014), as well as the principle sigma factor gene *sigA*, which has a putative Lsr2‐binding site in its promoter (Rustad *et al.*, 2014). However, expression of these genes did not significantly change in Δ*lsr2_T112A_* versus Δ*lsr2*_WT_ in accordance with our ChIP‐sequencing data.

### PknB‐mediated phosphorylation of Lsr2 or phosphomimetic mutation of Lsr2 reduced its DNA binding *in vitro*

**Figure 5:**
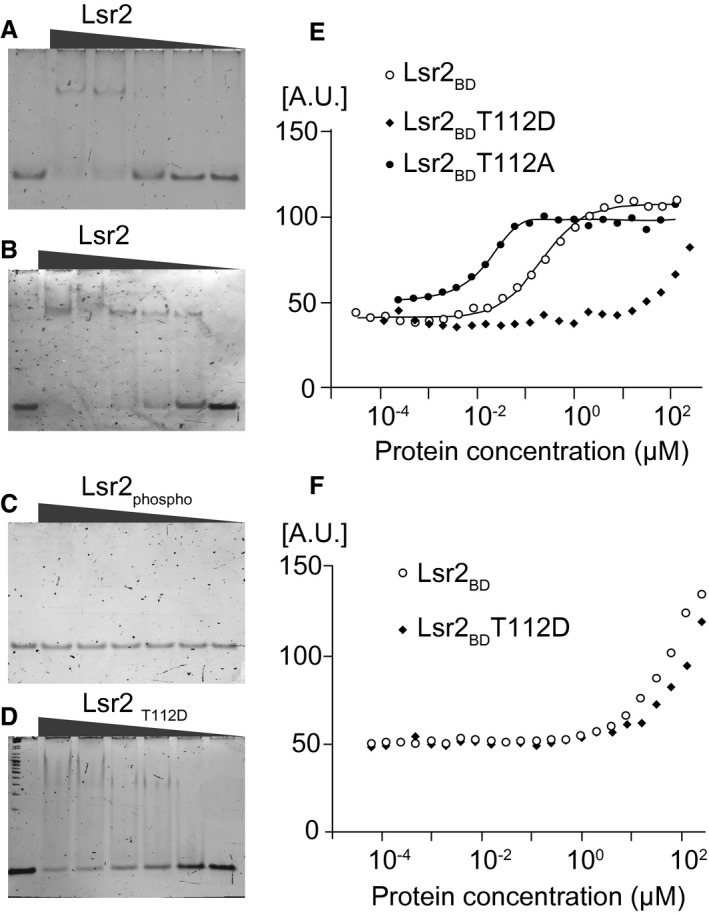
Phosphorylatied Lsr2 or its phosphomimetic T112D variant do not bind DNA. A. Lsr2 was mixed with *leuS* fragment containing a putative binding site (AATTCGGCAAAATCGGTAAG), position of which is marked with an asterisk in Fig. 4A. B. Lsr2 was mixed with the mutated *leuS*_MUT_ fragment (AACTCGGCGAGGTCGGTCAG). C. Lsr2 was phosphorylated by PknB and mixed with *leuS*‐binding site. D. Lsr2 T112D variant was mixed with *leuS* fragment. Lsr2 was added to DNA at a range of concentrations (0.95–7.6 μM). Representative results from three independent experiments shown. E. Quantification of Lsr2_BD_ (DNA‐binding domain) interaction with DNA by fluorescence anisotropy. Titration of 5’ Alexa Fluor 488 double‐stranded DNA (CGCGCATATATGCG) (4 nM) by Lsr2_BD_ WT (open circles), Lsr2_BD_ T112A (black circles) and Lsr2_BD_ T112D (black diamonds). F. control experiments with GC‐rich double‐stranded DNA fragment (AACTCGGCGAGGTCGGTCAG).

To determine how phosphorylation of Lsr2 affects the affinity and sequence specificity of DNA binding, we performed electrophoretic mobility shift assays (EMSA) with a range of DNA fragments containing putative Lsr2‐binding sites. These fragments were obtained by PCR or by annealing pairs of oligonucleotides to produce double‐stranded DNA (Table S2). Lsr2 reduced the mobility of all fragments when added at concentrations above 1.9 μM and there was no apparent difference in affinity between the DNA fragments (Fig. S3). The same pattern was observed when a shorter double‐stranded DNA containing a putative Lsr2‐binding site within the *leuS* gene (Reddy *et al.*, 2009) was used (Fig. 5A). We tested various DNA fragments, including a mutated *leuS* site (Fig. 5B), and all were shifted by Lsr2 regardless of their sequence, demonstrating that Lsr2 binds non‐specifically to DNA, as previously suggested (Colangeli *et al.*, 2007).

We next assessed the effect of Lsr2 phosphorylation on binding to these DNA fragments. As shown in Fig. 5C, phosphorylation of Lsr2 by PknB completely abolished binding (Figs 5C and S3), whereas Lsr2 T112D variant showed reduced DNA binding (Fig. 5D). This recalls the ChIP‐sequencing results where Lsr2 T112D and wild‐type Lsr2 (that is phosphorylated in cells), precipitated less DNA than phosphoablative T112A Lsr2.

*In vitro* phosphorylation of Lsr2 resulted in phosphorylation of both domains. Like Lsr2, HN‐S proteins also consist of an oligomerisation domain and DNA‐binding domain. In the HN‐S family, the two domains have distinct roles: shaping the nucleoid and regulating gene expression respectively (Winardhi *et al.*, 2015). In the case of Lsr2, threonine 112 in the DNA‐binding domain was required for function in *Mtb*, whereas other putative phosphorylation sites in the oligomerisation domain were mutated without loss of function (Fig. 3). To discriminate between the effects of phosphorylation on these two domains, we purified the DNA‐binding domain (Lsr2_BD_) to compare DNA binding of phosphorylated and unphosphorylated protein_._ A fluorescence anisotropy approach was used, since truncated Lsr2_BD_ was not suitable for EMSA.

Previous studies demonstrated that Lsr2_BD_ mainly recognised AT‐rich DNA sequences that formed a hook‐like structure (Gordon *et al*, 2011). We measured to ability of Lsr2_BD_ and T112 mutants to bind an Alexa 488N labelled AT‐rich double‐stranded DNA fragment (5′‐CGCGCATATATGCG‐3′) (Fig. 5E). At pH 7.5 the Kd value for Lsr2_BD_ was 0.21 ± 0.06 µM, that for Lsr2_BD_T112A was 0.02 ± 0.008 µM and Lsr2_BD_ T112D showed no significant DNA binding (Kd could not be determined). In control experiments, Lsr2_BD_ or Lsr2_BD_T112D showed no binding to a GC‐rich DNA sequence (Fig. 5F), confirming Lsr2_BD_ preferential binding to AT‐rich DNA. These results show that the T112D mutation, mimicking the Lsr2 phosphorylated state, reduced Lsr2_BD_T112D binding to DNA *in vitro*. In summary, phosphorylation controls both Lsr2 and Lsr2_BD_ binding to DNA.

### Phosphomimetic T112D variant changed the conformation entropy of the Lsr2 DNA‐binding domain

**Figure 6:**
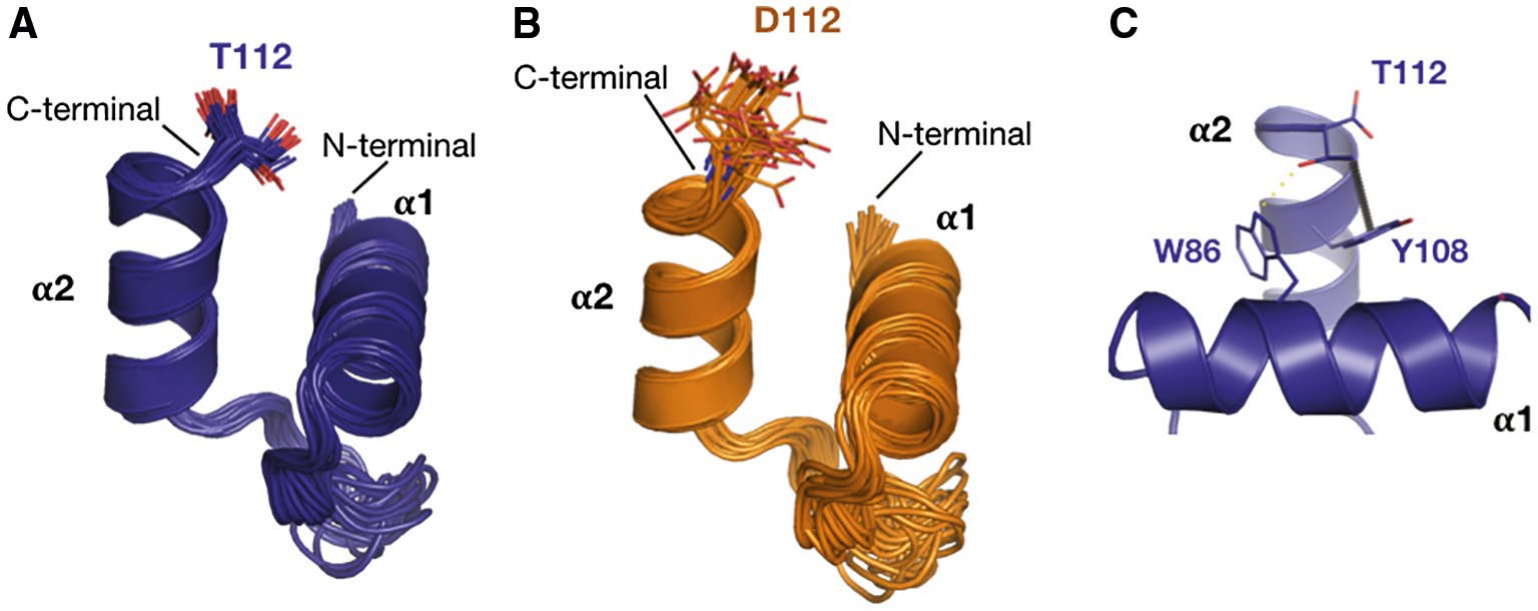
Solution structure of Lsr2_BD_ (A) and Lsr2_BD_T112D (B). Superimposition of the 20 best calculated structures in cartoon representation with the last residues represented as sticks (A and B). α1 helix and linker region are involved in DNA binding; the structure of these regions is not affected by T112D mutation. In Lsr2_BD,_ the threonine T112 side chain interacts with both tyrosine Y108 (3.7 Å) and tryptophan W86 (3.0 Å), all represented in sticks (C).

To elucidate a molecular basis for the reduced binding of phosphomimetic Lsr2 to DNA we compared nuclear magnetic resonance (NMR) structures of Lsr2_BD_WT and Lsr2_BD_T112D. In accordance with the previously published structure (Gordon *et al.*, 2010), our data confirmed that Lsr2 _BD_ consists of two perpendicular α‐helices (α1, residues 78–89; α2, residues 102–112) linked by a long loop (residues 90–101). The two major components involved in Lsr2 to DNA binding are residues Arg97–Gly98–Arg99 that are inserted into the minor grove of DNA and Arg77, Ser 80, Arg84 and Ser95 that interact with the phosphate‐sugar backbone of the minor grove (Gordon *et al.*, 2010). This organisation was preserved in Lsr2_BD_T112D (Table S6). However, T112D mutation resulted in a shorter α2 helix (Fig. S4), which ended with alanine 111 in Lsr2_BD_T112D compared with threonine 112 in Lsr2_BD_WT (Fig. 6A and B). Both variants were monomeric and the N‐terminal segment (residues 66‐75) upstream of the DNA‐binding domain was disordered. We also found that in Lsr2_BD_ the methyl group of threonine 112 interacted with tyrosine 108, while the hydroxyl group of threonine 112 interacted with tryptophan 86 (Fig. 6C). These interactions did not form in Lsr2_BD_T112D, likely accounting for the shorter helix. Lsr2_BD_ T112A, which must lack both interactions, bound DNA in our anisotropy experiments (Fig. 5E), demonstrating that the interactions themselves are not required for DNA binding, while suggesting that changes in conformation or dynamics related to the shorter helix could account for changes in DNA binding.

^15^N heteronuclear NMR relaxation analysis was performed to assess the dynamic behaviour of the two proteins (Fig. S5). In both isoforms, two α helices showed similar amplitudes for internal motion. However, in Lsr2_BD_ mainly the C‐terminal helix was affected, while in Lsr2_BD_T112D the internal motion was extended to the N‐terminal helix. A previous study on the catabolite activator protein (CAP) demonstrated that different protein mutants with the same structure of interaction interface displayed very different affinity for their target DNA (Tzeng and Kalodimos, 2012). The authors showed that changes of the binding affinity were linked to fast internal dynamics (conformation entropy). Similarly, the T112D mutation and, presumably, phosphorylation of threonine 112, resulted in a shorter α2 helix and a more mobile loop that increased the Lsr2_BD_ dynamics and impaired DNA binding.

## Discussion

*Mtb* can subvert the immune system to survive in the host for many years. This remarkable ability is determined by mechanisms that allow *Mtb* to respond to multiple environments and adjust metabolic activity and cell division. One of these regulatory systems is protein phosphorylation involving 11 serine/threonine kinases, including protein kinase B. PknB is expressed during active replication (Kang *et al.*, 2005) and is essential for growth in standard media (Fernandez *et al.*, 2006; Forti *et al.*, 2009). PknB has been implicated in the regulation of peptidoglycan biosynthesis (Bellinzoni *et al.*, 2019); its main substrate CwlM stimulates biosynthesis of peptidoglycan precursors (Boutte *et al.*, 2016) and may facilitate their transport to the cell surface (Turapov *et al.*, 2018). This regulation is crucial for adjusting bacterial growth and synthesis of the cell wall. The external PASTA domain of PknB (Barthe *et al.*, 2010; Prigozhin *et al.*, 2016) is essential for PknB‐mediated signalling and its disruption results in bacterial death and alteration of antimicrobial susceptibility (Chawla *et al.*, 2014; Turapov *et al.*, 2015; Turapov *et al.*, 2018). Furthermore, PknB can phosphorylate sigma factor SigH and its cognate anti‐sigma factor RshA; phosphorylation disrupts interaction of these two proteins and result in increased expression of the SigH regulon (Park *et al.*, 2008).

Here, we present data demonstrating that PknB also controls global gene expression via another substrate, the DNA‐binding protein Lsr2. Transcriptomic analysis of PknB‐depleted *Mtb* suggest that PknB phosphorylation may silence alternative pathways, which are not important for logarithmic growth but which may be critical for mediating stress responses and virulence. These include enzymes involved in alternative metabolic pathways, synthesis of complex lipids, regulators of stress responses and antimicrobial tolerance (Fig. 1, Table S1). PknB depletion resulted in the altered expression of several transcriptional regulators and the regulons of Rv0081, DosR and Lsr2. However only Lsr2 was more phosphorylated in PknB‐producing *Mtb* compared with PknB‐depleted *Mtb*. According to our transcriptomic and proteomic signatures, none of the other serine/threonine protein kinases were upregulated in these conditions (Table 1), suggesting that PknB was responsible for Lsr2 phosphorylation in growing *Mtb.*

We demonstrate that Lsr2 could be phosphorylated by PknB at several threonines but only the T112 was essential for growth and survival of *Mtb*. The phosphoablative T112A mutant could not complement the growth defect of the *Mtb lsr2* deletion mutant and was impaired in the Wayne hypoxia model. To investigate the molecular mechanism responsible for the growth defect in Δ*lsr2*_T112A_ we conducted ChIP‐Seq analysis and compared DNA‐binding patterns in Δ*lsr2*_T112A,_ Δ*lsr2*_T112D_ and Δ*lsr2*_WT_. In agreement with previously published data (Gordon *et al.*, 2010; Minch *et al.*, 2015) we detected multiple occurrences of DNA binding in all three Lsr2 backgrounds supporting both nucleoid shaping and gene regulatory functions of Lsr2. However, phosphoablative Lsr2 T112A variant had increased DNA binding, potentially directly affecting expression of 94 genes. Interestingly, most of these genes were not essential for growth *in vitro* (DeJesus *et al.*, 2017). Moreover, deletion of some of these genes has been previously shown to be advantageous for growth (DeJesus *et al.*, 2017), including genes of unknown function (*Rv0888*, *Rv1958*, *Rv1957*), PE/PPE genes (*Rv0878*, *Rv1983*), *aprA* and genes controlling transport of PDIM (*drrB* and *drrC*). While products of these genes might be disadvantageous for growth *in vitro*, they likely play a critical role in adaptations to stress and virulence (Camacho *et al.*, 2001; Sassetti and Rubin, 2003; Rohde *et al.*, 2007).

Our data demonstrate that PknB phosphorylation of Lsr2 *in vitro* completely abolished DNA binding, while the phosphomimetic mutation reduced Lsr2–DNA interactions (Fig. 5). We have not investigated the effect of T8, T22 and T31 phosphorylations on DNA binding. Based on previously published data we hypothesise that phosphorylation of these sites in the oligomerisation domain might be important for controlling nucleoid shape and DNA bridging properties. The results of our study suggest that phosphorylation of T112 in the DNA‐binding domain controls interaction of Lsr2 with AT‐rich DNA sequences which modifies gene expression (Fig. 4C). Our structural studies further confirm that phosphomimetic T112D Lsr2 variant had a shorter C‐terminal helix and increased dynamics of the DNA‐binding domain, leading to impaired Lsr2–DNA binding (Figs 6 and S5).

Post‐translational modifications are common mechanisms for the regulation of DNA binding both in eukaryotes (Bannister and Kouzarides, 2011) and prokaryotes (Dilweg and Dame, 2018). Phosphorylation or nitrosylation of transcriptional regulators abolish DNA binding (Leiba *et al.*, 2014; Smith *et al.*, 2017). H‐NS protein, a homologue of Lsr2 in *E. coli*, has been shown to be acetylated and phosphorylated; however, the precise function of these modifications remains to be characterised (Dilweg and Dame, 2018). Our data show that phosphorylation of Lsr2 is important for *Mtb* growth and that this may be a key mechanism for controlling mycobacterial adaptations to permissive and non‐permissive environments. Thus, PknB mediates two critical components of mycobacterial growth, peptidoglycan biosynthesis and gene expression of alternative pathways.

Based on our data, we propose that PknB controls *Mtb* growth by phosphorylating Lsr2. Like other H‐NS‐like proteins Lsr2 plays a dual role in mycobacterial biology, it shapes and protects the nucleoid and it controls gene expression (Bartek *et al.*, 2014; Kriel *et al.*, 2018). However, unlike H‐NS proteins in Gram‐negative bacteria that mainly silence the expression of foreign DNA (Lucchini *et al.*, 2006), Lsr2 regulates expression of genes that are essential for growth, virulence and adaptation (Bartek *et al.*, 2014). Our study suggests that phosphorylation of T112 might be important for tuning gene expression during growth, and the dynamic change between phosphorylated and non‐phosphorylated Lsr2 may help to adjust transcriptional patterns according to growth conditions. Reduced T112 phosphorylation, for example during starvation, may increase Lsr2 binding and upregulate pathways that are critical for *Mtb* survival under these conditions. Our data suggest that PknB is the main serine/threonine kinase responsible for phosphorylation of Lsr2 at T112 during growth, however we cannot exclude that other kinases can phosphorylate Lsr2 at this or other sites under different conditions as it has previously been shown for other substrates (Baer *et al.*, 2014).

While there are many outstanding questions on the precise mechanisms of PknB‐mediated regulation of gene expression and Lsr2 binding to DNA, our findings provide a functional link between serine/threonine protein kinase signalling and transcriptional regulatory pathways that enable *Mtb* to survive the varied environments encountered during infection.

## Experimental procedures

### Strains and media

*Mtb* H37Rv was grown in Middlebrook 7H9 **(**Becton, Dickinson and Company) liquid medium supplemented with 10% (v/v) Albumin–Dextrose Complex (ADC), 0.2% (v/v) glycerol and 0.1% (w/v) Tween 80 at 37°C with shaking or in SMM containing hygromycin with or without pristinamycin. SMM comprised of 0.3 M sucrose, 20 mM MgSO_4_, 0.1% Tween 80 (w/v), 10% (v/v) ADC in standard 7H9 broth. Bacterial growth was measured by absorbance at 580nm, or by colony‐forming unit (CFU) counting on 7H10 agar (Becton, Dickinson and Company), or by most probable number (MPN) counting using established protocols (Loraine *et al.*, 2016) and the MPN calculator program (Jarvis *et al.*, 2010). *Escherichia coli* OverExpress™ C41(DE3) and DH5α were grown in Lysogeny broth. For protein expression, *E. coli* was grown to mid‐log phase (OD_600_ 0.6‐0.8) at 37°C with shaking at 200 rpm before adding 0.5 mM isopropyl β‐D‐1‐thiogalactopyranoside followed by incubation at 18°C overnight. Antibiotics were used at the following concentrations (μg/ml): pristinamycin, 0.5; kanamycin, 50; hygromycin, 50; ampicillin, 50. Wayne model of non‐replicating persistence was set up as previously described (Wayne and Sramek, 1994). CFU and MPN counts were determined at 0‐, 7‐ and 12‐week time points.

### Genetic manipulations

Previously described *pknB*‐CM (Forti *et al.*, 2009) and Δ*lsr2* mutants (Bartek *et al.*, 2014) were used in this study. In Δ*lsr2* mutant, a gene fragment encoding the C‐terminal DNA‐binding domain (corresponding to 174–268 bp region) was deleted. The *Rv3597* (*lsr2*) coding sequence with additional 200 bp upstream region containing the putative promoter was amplified from the *Mtb*H37Rv genome using Platinum Taq‐HF polymerase (ThermoFisher) and cloned into the pMV306 plasmid that integrates at *attB* site of *Mtb* chromosome (primers in Table S2). Lsr2 *Mtb* variants were obtained using the GeneArt™ Site‐Directed Mutagenesis System (Thermo Fisher Scientific) according to the manufacturer's instructions. All constructs were sequenced before transformation into an *Mtb lsr2* deletion mutant. Transformants were selected on 7H10 agar or in supplemented 7H9 containing kanamycin. For expression of Lsr2 proteins *Mtb* H37Rv *lsr2*‐coding sequence or shorter fragments were PCR amplified and cloned into pET15‐TEV and the resultant constructs were transformed into *E. coli* OverExpress™ C41 (DE3). Bacterial strains generated in this study are shown in Table S2.

### Preparation of samples for proteomics and transcriptomics

*Mtb pknB*‐CM was grown to OD_580nm_ ~0.7 in SMM with or without pristinamycin. For proteomics studies, washed *pknB*‐CM bacteria were resuspended in buffer containing 20 mM TrisCl, pH 7.5, 1 M NaCl, 8 M urea and proteinase/phosphatase inhibitors. After bead beating, lysates were cleared by centrifugation and filtration and treated using the FASP protocol, as described previously (Turapov *et al.*, 2018; Iswahyudi *et al.*, 2019). For transcriptomics analysis, bacterial cultures were incubated with four volumes of guanidine thiocynate (GTC) for 30 minutes prior centrifugation.

### Quantitative label‐free proteomics analysis

Analysis was performed as previously described (Turapov *et al.*, 2018; Iswahyudi *et al.*, 2019). For quantification, all peptides of an identified protein were included and the total cumulative abundance was calculated by summing the abundance of all peptides allocated to the respective protein. Additionally, Scaffold Q+ (version Scaffold_4.3.4, Proteome Software Inc) was used for peptide and protein identifications as previously described (Turapov *et al.*, 2015). Peptide probabilities from Mascot were assigned by the Scaffold Local FDR algorithm.

### Transcriptomic analyses

RNA was extracted from three biological replicates using the GTC/Trizol method (Waddell and Butcher, 2010). *Mtb* RNA (2 μg) was enzymatically labelled with Cy3 fluorophore and hybridised to a *Mtb* complex microarray (ArrayExpress accession number A‐BUGS‐41) as previously described (Salina *et al.*, 2014). For quantitative RT‐PCR, total RNA was isolated from triplicate *Mtb* cultures; cDNA was generated using Superscript Reverse Transcriptase II and mycobacterial genome‐directed primers (Rachman *et al.*, 2006). qPCR was performed in a Corbett Rotor Gene 6000 real‐time thermocycler using Absolute qPCR SYBR Green mix and gene expression values were normalised to 16S rRNA expression. For qPCR three biological and two replicates were assessed.

### ChIP‐Seq analysis

DNA–Lsr2 interactions in Δ*lsr2*_T112A,_ Δ*lsr2*_T112D,_ Δ*lsr2*_WT_ were assayed using ChIP‐seq methods as previously described (Minch *et al.*, 2015). Briefly, mid log‐phase *Mtb* H37Rv cultures (OD_600_ 0.4–0.6) were crosslinked with 1% formaldehyde, followed by incubation with 125 mM glycine for 5 minutes at 37°C. The cells were mechanically lysed and then sonicated to produce 200–500 bp fragments. Input control samples were taken for each genotype before antibody was added to assess antibody specificity. Samples were immunoprecipitated using a polyclonal anti‐rabbit anti‐Lsr2 antibody and protein‐G agarose beads. The Lsr2 complexes were de‐crosslinked by heating at 65°C overnight and proteins removed by treatment with proteinase K (10 mg/ml) for 2 h at 55°C. The DNA samples were column‐purified (Qiagen) and the quality of purified IP‐Lsr2 DNA verified using the Qubit DNA HS quantification assay and Nanodrop spectrophotometer. Libraries were prepared and sequenced using Illumina HiSeq SE50, 20 million reads (Novogene, Hong Kong). Raw fastq files were aligned to the *Mtb* H37Rv (NC_000962.3) reference genome using bwa samse (Li and Durbin, 2009). MACS2 version 2.1.1.20160309 (Zhang *et al*., 2008) was used to compare each of the input controls to the immunoprecipitated samples, identifying Lsr2‐binding sites (callpeak) using default parameters but including ‘‐g 4.41e+06 –nomodel –extsize 147’ (Table S3). Differential peaks comparing Δ*lsr2*_T112A_ to Δ*lsr2*_WT_ were then identified using ‘macs2 bdgdiff’.

### Purification of recombinant proteins and phosphorylation *in vitro*

Recombinant Lsr2 proteins were purified using immobilised metal affinity chromatography (Ni‐NTA agarose, Qiagen) and size exclusion chromatography. The recombinant catalytic domain of PknB was purified using Glutathione Sepharose 4B GST‐tagged protein purification resin (GE Healthcare). For phosphorylation recombinant Lsr2 (10 μM) was mixed with the recombinant catalytic domain of PknB (5 μM) in a kinase buffer (20 mM Tris–HCl, pH 8.0; 0.5 mM DTT; 10 mM MgCl_2_; 0.2 mM ATP) and incubated at 37°C for 1h. Phosphorylation was confirmed by western blot analysis. Phosphorylated residues were identified in trypsin‐digested proteins using LTQ‐Orbitrap‐Velos mass spectrometer.

### Protein electrophoresis and western blotting

Proteins were separated on 4–20% gradient SERVA gels and transferred onto a nitrocellulose membrane using a Trans‐Blot® Turbo™ Transfer System (Bio‐Rad). SIGMAFAST™ BCIP®/NBT or SignalFire™ Elite ECL Reagent were used to visualise proteins on C‐DiGit Chemiluminescent Blot Scanner (LI‐COR Biosciences). The following antibodies were used: custom polyclonal antibody raised against Lsr2 in rabbit (Gemini Biosciences); monoclonal murine anti‐polyhistidine antibody (Sigma‐Aldrich); phospho‐threonine antibody (Cell Signaling Technology); monoclonal anti‐*Mtb*GroEL2 (*Rv0440*), clone IT‐70 (BEIResources); mouse anti‐rabbit IgG antibody:alkaline phosphatase (Sigma‐Aldrich; anti‐mouse IgG (whole molecule:alkaline phosphatase antibody produced in rabbit (Sigma‐Aldrich), and anti‐rabbit IgG, HRP‐linked antibody (Cell Signaling Technology).

### Electrophoretic mobility shift assay

Electrophoretic mobility shift assays were carried out with DNA fragments amplified by PCR or annealed oligonucleotides (see Table S2 for primer details). DNA (1.2 nM) was mixed with indicated amounts of Lsr2 in a total volume of 20µl reaction buffer containing (10 mM Tris–HCl, pH 7.5, 50 mM KCl, 1 mM DTT, 5 mM MgCl_2_, and 2.5% glycerol). The mixture was incubated for 30 min at room temperature followed by native polyacrylamide gel electrophoresis using 8% gels in 0.5 x Tris‐Borate‐EDTA buffer, pH 7.5 for 24 min at 120 V. The gels were stained with SYBR Safe DNA stain (Thermo Fisher Scientific) and visualised using a ChemiDoc system (Bio‐Rad).

### Fluorescence anisotropy

Custom made 5′ Alexa Fluor 488 succinimidyl ester labelled oligonucleotide probe (sequence 5′‐CGCATATATGCGCG‐3) was purchased from Integrated DNA Technologies. Steady‐state fluorescence anisotropy‐binding titrations were performed on a Tecan Saphire II microplate reader, using a 470 nm LED for excitation and a monochromator set at 530 nm (bandwidth 20 nm) for emission.

### Determination of solution structures and dynamics of Lsr2_BD_ and Lsr2_BD_T112D

All ^1^H‐^15^N double‐resonance NMR experiments were performed at 20°C on Bruker Avance III spectrometers (700 or 800 MHz) using previously described methods (Barthe *et al.*, 1999; Gordon *et al.*, 2010). NMR samples of 0.5mM ^15^N‐labelled protein dissolved in 25mM sodium phosphate buffer (pH 6.8), 150mM NaCl with 10% D_2_O for the lock. ^1^H chemical shifts were directly referenced to the methyl resonance of DSS, while ^15^N chemical shifts were referenced indirectly to the absolute ^15^N/^1^H frequency ratio. All NMR spectra were processed with GIFA (Pons *et al*, 1996). Chemical shift assignments were made using standard NOESY, TOCSY experiments performed on the ^15^N‐labelled protein sample. NOE cross‐peaks identified on 3D [^1^H, ^15^N] NOESY‐HSQC (mixing time 160 ms) were assigned through automated NMR structure calculations with CYANA 2.1 (Güntert, 2004). Backbone ϕ and φ torsion angle constraints were obtained from a database search procedure on the basis of backbone (^15^N, HN, Hα) chemical shifts using TALOS+ (Shen *et al.*, 2009). For each protein, a total of 200 three‐dimensional structures were generated using the torsion angle dynamics protocol of CYANA 2.1. The 20 best structures of each protein (based on the final target penalty function values) were minimised with CNS 1.2. All statistical parameters are summarised in (Table S6). Relaxation rate constant measurements were performed on a 0.5mM protein sample, at 18.8 T (800 MHz). The pulse sequences used to determine ^15^N R_N_(N_z_) (R_1_), R_N_(N_xy_) (R_2_), and ^15^N{^1^H} NOE values were similar to those described (Barthe *et al.*, 1999). The ^15^N longitudinal relaxation rates (R_N_(N_z_)) were obtained from 10 standard inversion recovery experiments, with relaxation delays ranging from 18 to 1206 ms. The ^15^N transverse relaxation experiments (R_N_(N_xy_)) were obtained from 10 standard CPMG experiments, with relaxation delays ranging from 16 to 160 ms. Both series of experiments were acquired in two single interleaved matrices to ensure uniformity of the experimental conditions. Heteronuclear ^15^N{^1^H} NOE were determined from the ratio of two experiments, with and without saturation.

### Statistical analysis

Calculation of the protein p‐values was performed on the sum of the normalised abundance across all runs using one‐way ANOVA. Significantly differentially expressed genes (Table S1) were identified using a moderated *t*‐test (*P*‐value < 0.05 with Benjamini and Hochberg multiple testing correction), and fold change> 1.8 in GeneSpring 14.5 (Agilent Technologies). Hypergeometric probability and TFOE analysis (Rustad *et al.*, 2014) were used to identify significantly enriched signatures. An unpaired *t*‐test was performed to compare gene expression in Δ*lsr2*_WT_ and Δ*lsr2*_T112A_. For evaluation of growth parameters and survival in the Wayne model, one‐way ANOVA (GraphPad Prism) was used, comparing Δ*lsr2*_pMV_ or Δ*lsr2*_T112A_ to Δ*lsr2*_WT_ and Δ*lsr2*_T112D_.

## Author contributions

Conceptualisation, OT, MCG, SJW and GVM; Methodology, ARB, PA, HJ, CR, AAW, MW; Investigation KA, OT, PB, HJ, ADV, GVM; Analysis, OT, AAW, KA, HJ, MCG, SJW, GVM; Resources, MW, ILB and MIV; Writing – Original Draft, GVM, MCG, SWJ; Writing – Review and Editing, GVM, SJW, MIV, HMO. Funding Acquisition, KA, MCG, SJW, GVM. Supervision, GVM, OT, HMO, MCG and SJW.

## Conflict of interest

Paul Ajuh is the director and shareholder in Gemini Biosciences Limited, Liverpool, UK. Other authors declare no competing interests.

## Data Availability

Database: Mycobrowser release 3 2018‐06‐05, 8 ://mycobrowser.epfl.ch (Kapopoulou *et al.*, 2011). The accession numbers for the mass spectrometry proteomics data reported in this paper are ProteomeXchange Consortium via the PRIDE partner repository PXD009239 and 10.6019/PXD009239 (://www.proteomexchange.org). The accession numbers for microarray data – ArrayExpress, E‐MTAB‐7627, ://www.ebi.ac.uk/arrayexpress; ChIP‐Seq datasets – the European Nucleotide Archive (ENA), PRJEB31102, ://www.ebi.ac.uk/ena/data/view/PRJEB3110; for Lsr2 protein structures – the Protein Data Bank ://www.wwpdb.org, PDB6QKP and PDB6QKQ; the chemical shifts – the BMRB, BMRB ID 34358 and BMRB ID 34358.
