# Supplementary material for: Protein kinase B controls Mycobacterium tuberculosis growth via phosphorylation of the transcriptional regulator Lsr2 at threonine 112
Source: Mol Microbiol. 2019 Oct 10;112(6):1847–62. doi: 10.1111/mmi.14398 (PMC6906086; doi:10.1111/mmi.14398)
Supplement: Supplementary file 1 [file MMI-112-1847-s001.pdf]

Protein kinase B controls *Mycobacterium tuberculosis* growth via phosphorylation of the transcriptional regulator Lsr2 at threonine 112

Kawther Alqaseer[1,2$], Obolbek Turapov[1$], Philippe Barthe[3], Heena Jagatia[4], Angélique De Visch[3], Christian Roumestand[3], Malgorzata Wegrzyn[5], Iona L. Bartek[6], Martin I. Voskuil[6], Helen M. O'Hare[1,7], Paul Ajuh[8], Andrew R. Bottrill[9], Adam A. Witney[10], Martin Cohen-Gonsaud[3*], Simon J. Waddell[4*], Galina V. Mukamolova[1*]

[1]Leicester Tuberculosis Research Group, Department of Respiratory Sciences, University of Leicester, Leicester, LE2 9HN, UK; [2]Department of Basic Science, Faculty of Nursing, University of Kufa, P.O. Box 21, Kufa, Najaf Governorate, Najaf, Iraq; [3]Centre de Biochimie Structurale, CNRS, INSERM, University of Montpellier, 34090 Montpellier, France; [4]Wellcome Trust Brighton and Sussex Centre for Global Health Research, Brighton and Sussex Medical School, University of Sussex, Brighton, BN1 9PX, UK; [5]Core Biotechnology Services, University of Leicester, University Road, Leicester, LE1 7RH, UK; [6]Department of Immunology and Microbiology, University of Colorado School of Medicine, Aurora, CO 80045, USA; [7]LISCB, Department of Molecular and Cell Biology, University of Leicester, University Road, Leicester, LE1 7RH, UK, [8]Gemini Biosciences Ltd, Liverpool Science Park, Liverpool, L7 8TX, UK; [9]Protein Nucleic Acid Laboratory, University of Leicester, Leicester, LE1 7RH, UK; [10]Institute for Infection and Immunity, St George's University of London, London, SW17 0RE, UK.

* To whom correspondence should be addressed. E-mail: gvm4@le.ac.uk; martin@cbs.cnrs.fr; s.waddell@bsms.ac.uk.

[$]Equally contributed to this work.

Supplementary figures 1-5

Supplementary tables 2, 6

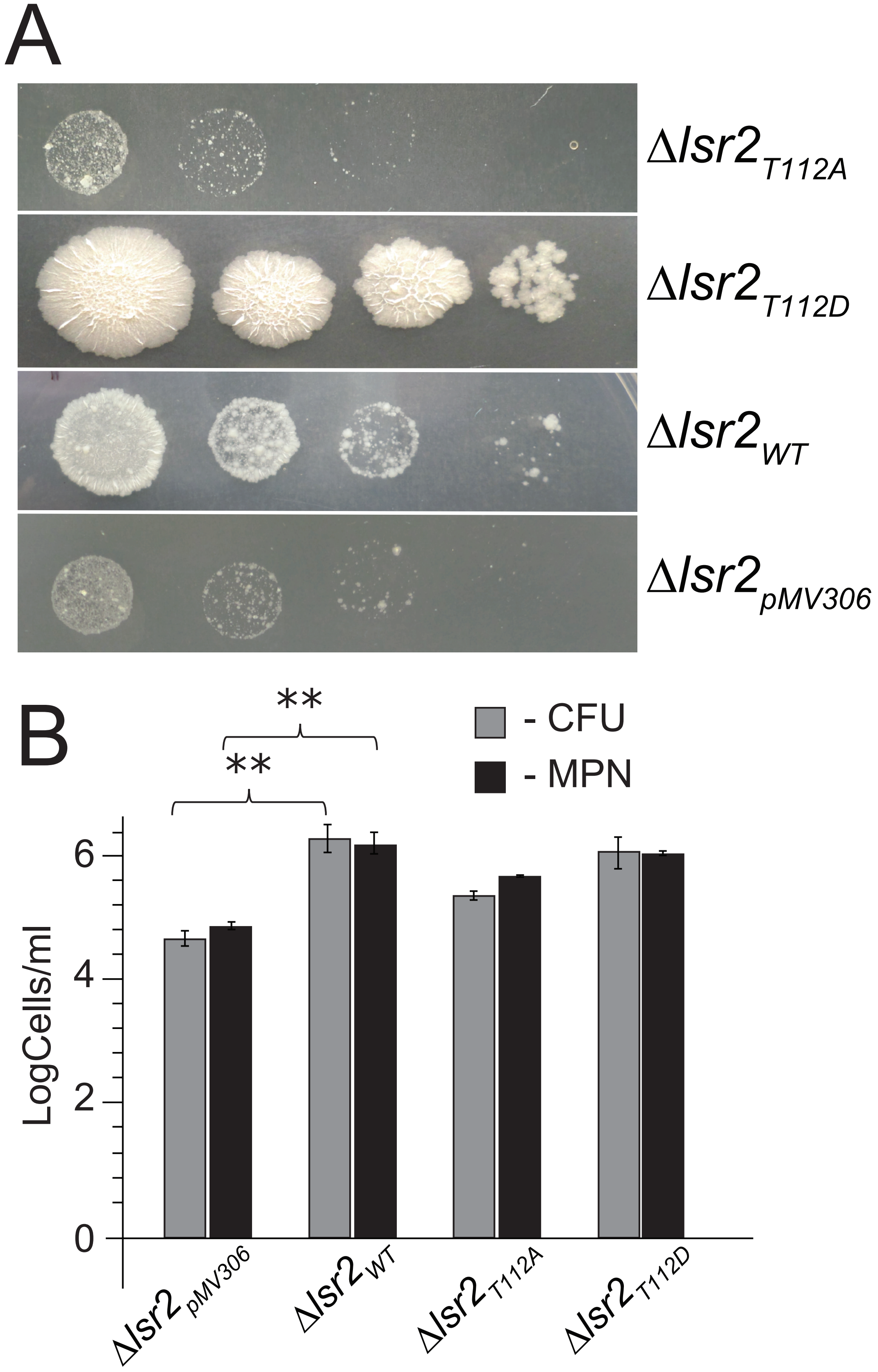

Figure S1. Effect of Lsr2 mutations on growth on agar and survival in prolonged stationary phase. (A) Lsr2 phosphoablative mutants were serially diluted and plated on 7H10 agar. (B) *Mtb*Lsr2 mutants were incubated with shaking for up to 42 weeks. Viable counts were determined by CFU and MPN counting. Represented as mean ± SEM (N=6).

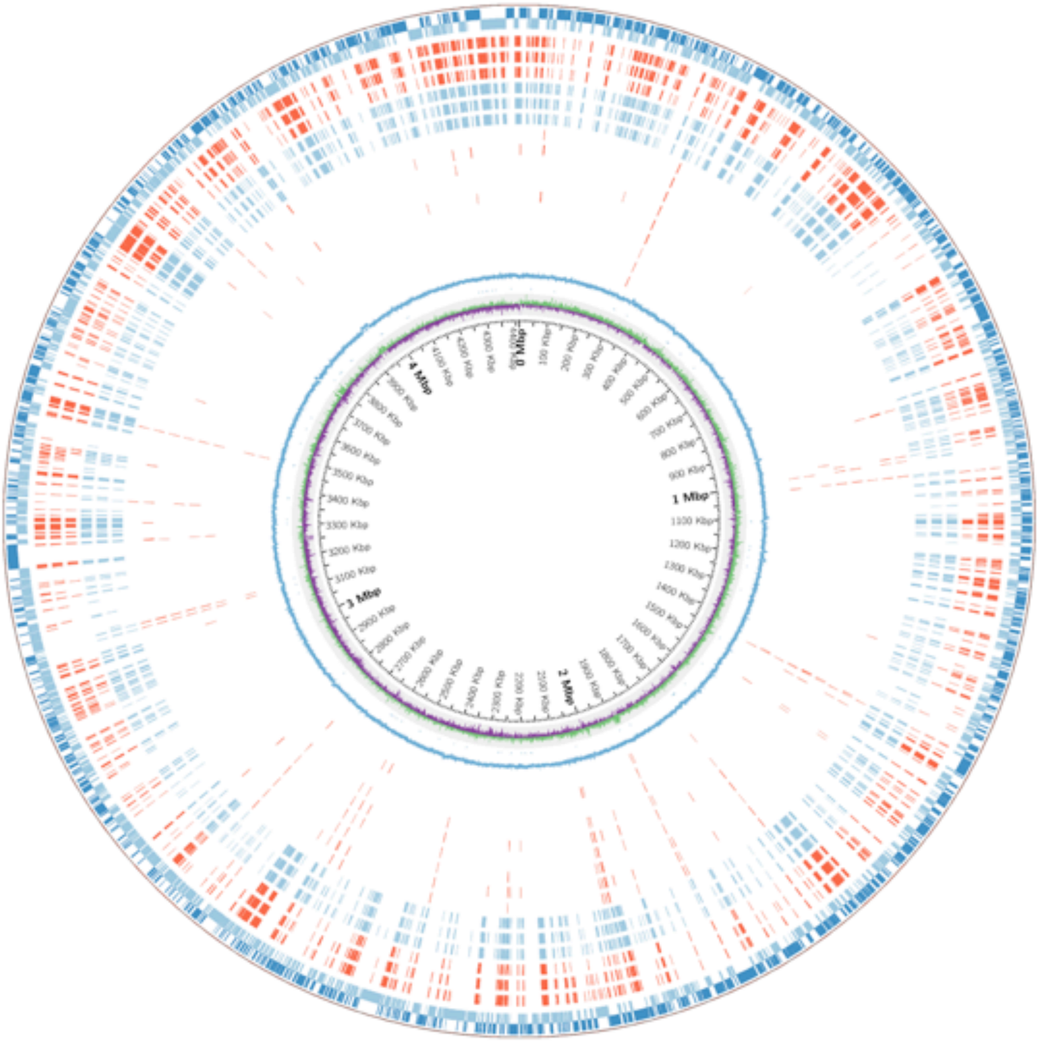

Figure S2. The genome-wide binding pattern of Lsr2 in *Mtb*, showing the impact of phosphorylation on binding. Moving from outside to inside, rings show the forward and reverse strands of the *Mtb* genome (blue), wild type Lsr2 binding events in 3 biological replicates (red), phosphoablative (T112A) Lsr2 binding sites in 3 biological replicates (blue), increase in abundance of Lsr2 binding in phosphoablative compared to wild type Lsr2 from pairwise comparison of biological replicates (red), GC% (blue), and GC skew (green/purple). These genes, potentially directly regulated by Lsr2, significantly overlapped with previously identified Lsr2 binding patterns from three independent studies, as indicated by hypergeometric p-values $1.36x10^{-226}$ (Gordon et al., 2010), $2.05x10^{-190}$ (Reddy et al., 2009), and $1.74x10^{-84}$ (Minch et al., 2015), respectively. Interestingly, gene expression signatures associated with inactivation of Lsr2 (Bartek et al., 2014) were significantly enriched (hypergeometric probability $2.45x10^{-27}$), providing further evidence that Lsr2 may directly regulate gene expression. Genes repressed in response to macrophage infection $3.02x10^{-14}$ (Schnappinger et. al, 2003), sputum environment $8.70x10^{-18}$ (Garton et al., 2008) and acid- nitrosative stress $8.59x10^{-15}$ (Cossu et al., 2013) also significantly overlapped with the predicted Lsr2 regulon, suggesting that Lsr2 may regulate *Mtb* adaptations to the changing environment.

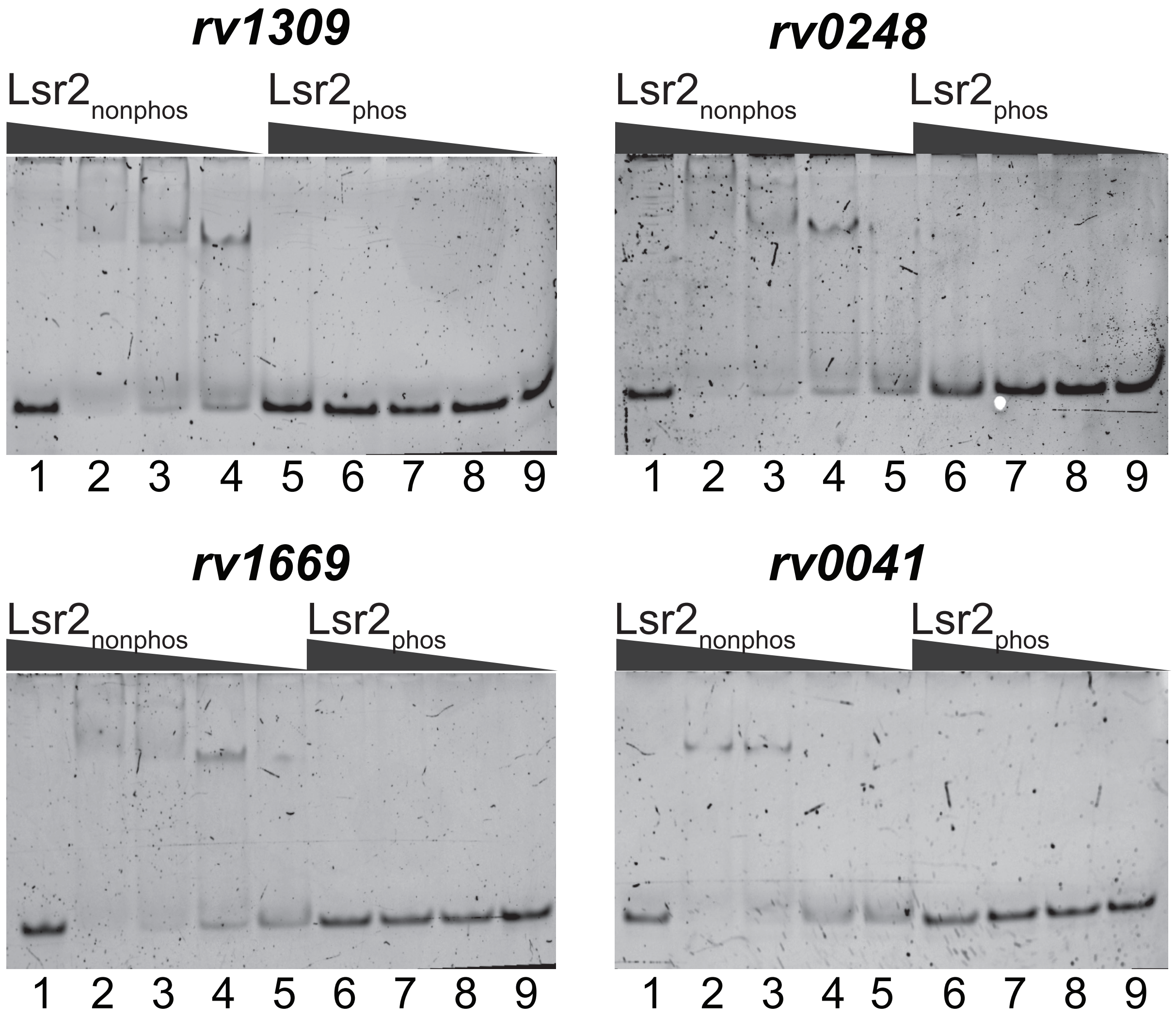

Figure S3. Phosphorylated and non-phosphorylated Lsr2 can shift various DNA fragments in EMSA. Lsr2 was mixed with annealed oligonucleotides containing putative binding sites within or upstream corresponding genes: *rv0041*, *rv0248*, *rv1306* and *rv1669.*

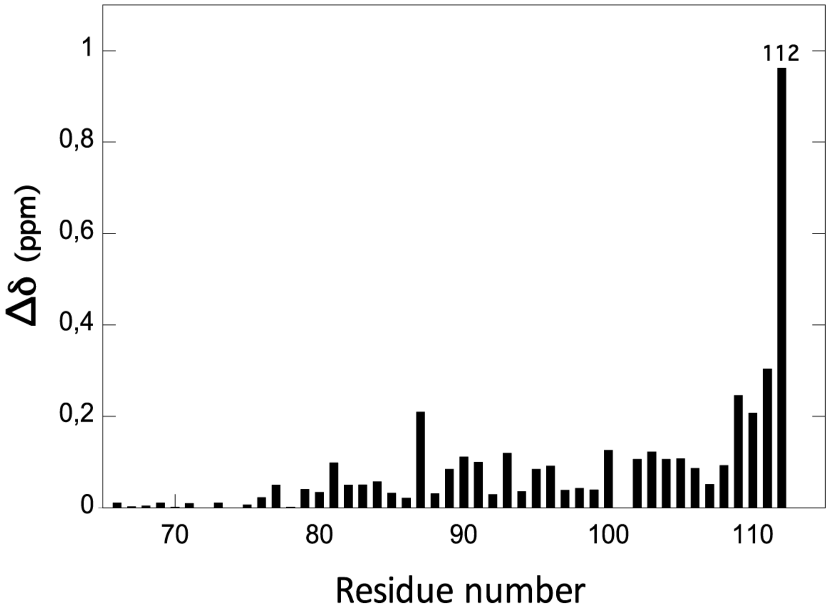

Figure S4. Amide averaged chemical shift variations (∆∂) as a function of the protein sequence.

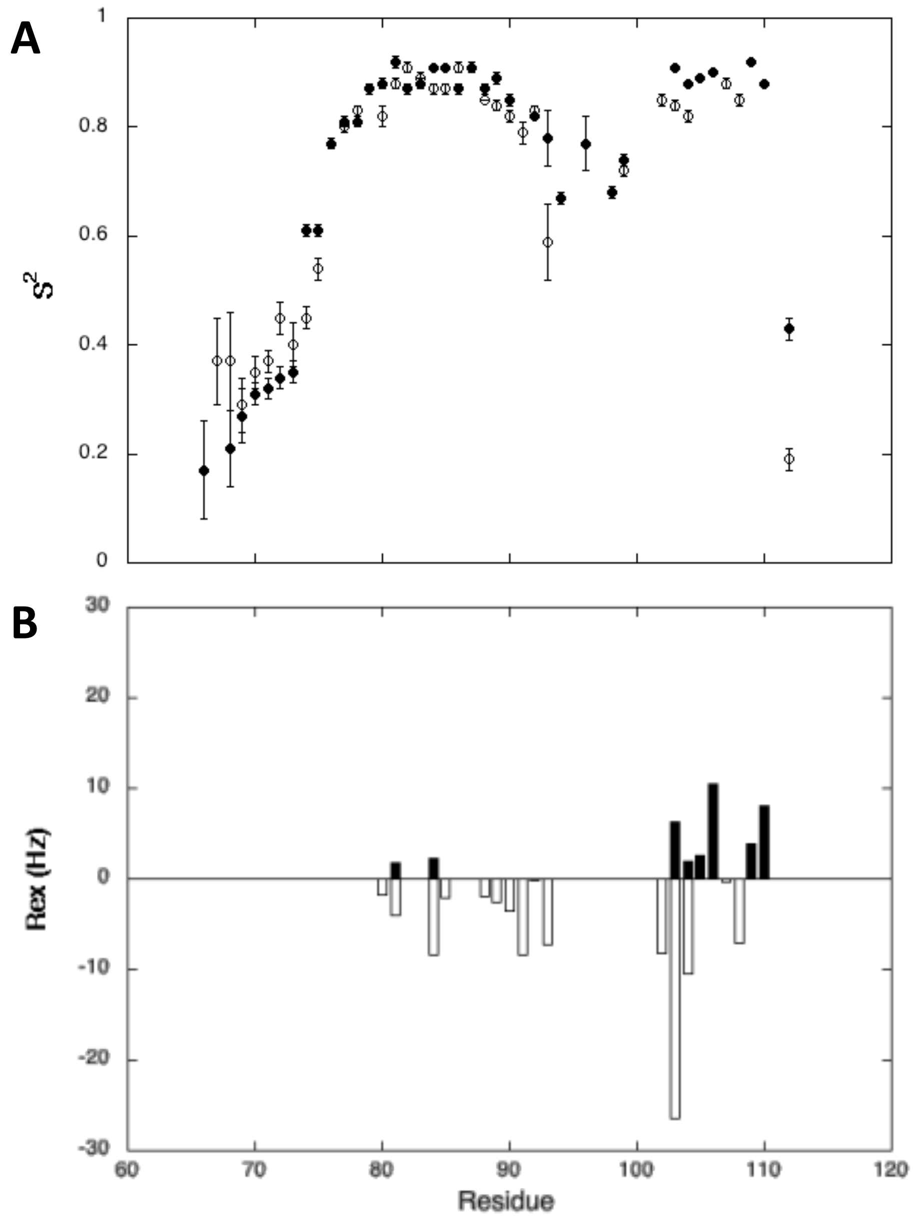

Figure S5. Model-Free Dynamic Analysis of $Lsr2_{BD}$ WT and $Lsr2_{BD}$T112D. (A) Generalized order parameters (S^2) values calculated for $Lsr2_{BD}$ (bold circles) and $Lsr2_{BD}$T112D (open circles). (B) Exchange contributions measured for $Lsr2_{BD}$ (bold bars) and $Lsr2_{BD}$T112D (open bars). For the sake of clarity, R_{ex} values are reported on a negative axis for $Lsr2_{BD}$T112D. The global correlation time (3.90 ± 0.2 ns) extracted from Lipari-Szabo "Model Free" analysis of the ^{15}N heteronuclear relaxation data (T_1, T_2, nOe) recorded on $Lsr2_{BD}$WT and $Lsr2_{BD}$T112D was consistent with a monomeric state for both proteins in our experimental conditions.

Supplementary tables

Table S1. The transcriptional signature of PknB-depletion in *Mtb*, comparing pristinamycin-inducible PknB complemented strains in sucrose magnesium medium with and without pristinamycin.

A separate excel file

Significantly differentially expressed genes were identified using a moderated t-test (p-value <0.05 with Benjamini and Hochberg multiple testing correction) and fold change >1.8 from three biological replicates. 65 genes were significantly induced and 34 genes were repressed by PknB-depletion in replicating *Mtb.*

Table S2. Strains and primers used and generated in the study.

Strains

Strain name	Strain description	Plasmid	Comments
***Mycobacterium tuberculosis* H37Rv strains**			
pknB-CM	PknB conditional mutant	pAZI9479::*pknB*	PknB depletion
Δ*lsr2*	Deletion mutant	None	Lsr2 mutant background
Δ*lsr2*$_{pMV}$	Empty plasmid control	pMV306	Δ*lsr2* complementation
Δ*lsr2*$_{WT}$	Δ*lsr2* complemented with wild type *lsr2*	pMV306::*lsr2*	Δ*lsr2* complementation
Δ*lsr2*$_{T8A}$	T8A Lsr2 variant	pMV306::*lsr2T8A*	Δ*lsr2* complementation
Δ*lsr2*$_{T22A}$	T22A Lsr2 variant	pMV306::*lsr2T22A*	Δ*lsr2* complementation
Δ*lsr2*$_{T31A}$	T31A Lsr2 variant	pMV306::*lsrT31A*	Δ*lsr2* complementation
Δ*lsr2*$_{T112A}$	T112A Lsr2 variant	pMV306::*lsr2T112A*	Δ*lsr2* complementation
Δ*lsr2*$_{T112D}$	T112D Lsr2 variant	pMV306::*lsr2T112D*	Δ*lsr2* complementation
***Escherichia coli* strains**			
C41(DE3) pET Lsr2	6xHis-Lsr2 expression strain	pET15bTEV::*lsr2*	Recombinant Lsr2
C41 (DE3) pET Lsr2 T112D	6xHis-Lsr2 T112D expression strain	pET15bTEV::*lsr2*$_{T112D}$	Recombinant Lsr2 T112D
C41 (DE3) pET Lsr2$_{BD}$	6xHis-Lsr2 DNA binding domain expression strain	pET15bTEV::*lsr2*$_{BD}$	Recombinant Lsr2$_{BD}$
C41 (DE3) pET Lsr2$_{BD}$T112D	6xHis-Lsr2 DNA binding domain T112D expression strain	pET15bTEV::*lsr2*$_{BD}$ *T112D*	Recombinant Lsr2$_{BD}$ T112D
C41 (DE3) pET Lsr2$_{BD}$ T112A	6xHis-Lsr2 DNA binding domain T112A expression strain	pET15bTEV::*lsr2*$_{BD}$ *T112A*	Recombinant Lsr2$_{BD}$T112A

BL21 (DE3) pGEX PknB	GST-PknB kinase domain expression strain	pGEX::*pknB kinase*	Recombinant PknB kinase domain

Primers

Primer name	Primer sequence (5'-3')	Comment
Lsr2testF	GTTGTGTCTGGATTGAGT	Lsr2 deletion confirmation
Lsr2testR	AAACCACCCAAGCGTTTC	Lsr2 deletion confirmation
pMV306lsr2F	CAC*GGTACC*GGAATGGGTATCGA	*Δlsr2* complementation
pMV306lsr2R	GACAAGCTTTCAGGTCGCCGCGT	*Δlsr2* complementation
pMV306lsr2AR	GACAAGCTTTCAGGCCGCCGCGT	*Δlsr2* complementation
pMV306lsr2DR	GACAAGCTTTCAGTCCGCCGCGT	*Δlsr2* complementation
T8AF	GCGAAGAAAGTAACCGTCGCCTTGGTCG ACGATTTCGAC	Lsr2 SDM
T8AR	GTCGAAATCGTCGACCAAGGCGACGGTT ACTTTCTTCGC	Lsr2 SDM
T22AF	TCGGGCGCCGCCGACGAAGCGGTCGAA TTCGGGCTTGAC	Lsr2 SDM
T22AR	GTCAAGCCCGAATTCGACCGCTTCGTCG GCGGCGCCCGA	Lsr2 SDM
T31AF	TTCGGGCTTGACGGGGTGGCCTATGAGA TCGACCTTTCC	Lsr2 SDM
T31AR	GGAAAGGTCGATCTCATAGGCCACCCCG TCAAGCCCGAA	Lsr2 SDM
Lsr-pETF	CAGCATATGGCGAAGAAAGTAACCGT	Recombinant Lsr2
Lsr-pETR	CGACTCGAGTCAGGTCGCCGCGTGGT	Recombinant Lsr2
Lsr-pETAR	CGACTCGAGTCAGGCCGCCGCGTGGT	Recombinant Lsr2 T112A
Lsr-pETDR	CGACTCGAGTCAGTCCGCCGCGTGGT	Recombinant Lsr2 T112D
pMV306F	TGGTATCTTTATAGTCCTGTC	pMV306 primer
pMV306R2	TAGTTAACTACGTCGACATCGA	pMV306 primer
LeuSsiteF	AATTCGGCAAAATCGGTAAG	EMSA
LeuSsiteR	CTTACCGATTTTGCCGAATT	EMSA
Rv0248csiteF	GTCTTCCAATTGTCCTTCGG	EMSA
Rv0248csiteR	CCGAAGGACAATTGGAAGA	EMSA
Rv1306siteF	TGTGGCGATTTATCGTGCCG	EMSA
Rv1306siteR	CGGCACGATAAATCGCCACA	EMSA
Rv1669siteF	CTACCACATTAATCGGCATC	EMSA
Rv1669siteR	GATGCCGATTAATGTGGTAG	EMSA
LeuSsmutF1	AACTCGGCGAGGTCGGTCAG	EMSA
LeuSsmutR1	CTGACCGACCTCGCCGAGTT	EMSA
Rv0192F1	AACACCGCGGTAAACATCGATGC	EMSA
Rv0192R1	TACGTCGAGGAGTCCATGACCAC	EMSA
leuSF1	CATTTGGTCTACCGATCGTGGAAG	EMSA
LeusR1	CTGTCATAGACGATCGGGAATGGT	EMSA
LeuSF2	CACGTCAGCTCTCGCGAGCCTTAC	EMSA
leuSR2	GGTGTGCTCGTCGACGACCAAGC	EMSA
leuS F	AGCCAACGTCGTCAACTT	qRT-PCR
leuSR	ATCCACTGAGTCCACCTGTA	qRT-PCR
sodAF	CACGTCAATCACACCATCTG	qRT-PCR
sodAR	GCACGGAACTTGTCGAAC	qRT-PCR
sigAF	GAG ATC GGC CAG GTC TAC GGC GTG	qRT-PCR

sigAR	CTG ACA TGG GGG CCC GCT ACG TTG	qRT-PCR
rpfAF	CTTGCAGTTCACTCAAAGCAC	qRT-PCR
rpfAR	CTCACCGACGGCAATCTG	qRT-PCR
rpfCF	AGCTGCCTCTCGGGAACAA	qRT-PCR
rpfCR	GACCACAGTGCGATCGGAAGG	qRT-PCR
16s rRNAF	TCCGGGCCTTGTACACA	qRT-PCR
16s rSRNAR	AACACCCGAAGCCAGTGG	qRT-PCR
icl1F	GCGGTGCGGAGGTGCTGTGG	qRT-PCR
Icl1R	AGGCTCTGGTCGGGGTAGGTG	qRT-PCR
ppsAF	ATC ATC GCC AAC CGC CTC TCG T	qRT-PCR
ppsAR	AAT ACC GCC GGG GAC AAC AAC AAA	qRT-PCR
nuoBF	CGA GAA GGT GGC GGG CTA TGT – 3’	qRT-PCR
nuoBR	GAA CCG CGC AAT GTC AAA CCT	qRT-PCR
tgs1F	AAC GAA GAC CAG TTA TTC GAG	qRT-PCR
tgs1R	CTC ATA CTT TCA TCG GAG AGC	qRT-PCR
RTPknBF1	TCAGAACGGAATCATCCACCGTGA	qRT-PCR
RTPknBR1	GCGATGCCGAAATCCATCACCTTT	qRT-PCR

Table S3. WT Lsr2 and phosphoablative Lsr2 (T112A) binding events measured by ChIP-seq in three biological replicates showing start and stop positions and binding event length.

A separate excel file

Average number of 690 binding events for wt Lsr2, median length 413 nt. Average number of 698 binding events for T112A Lsr2, median length 453 nt.

Table S4. The **Lsr2 regulon.**

A separate excel file

Putative regulons for wild type Lsr2 mapped to the *Mtb* H37Rv genome (Mycobrowser release 3 2018-06-05, 8). Individual ChIP-seq replicates are shown; the Lsr2 regulon was defined to have binding events in 2 of 3 biological replicates. Genes were included in the predicted regulon if the Lsr2 binding site was immediately upstream of, or within, the coding sequence of each gene. For Lsr2 intergenic binding between divergent genes, both genes were reported. For Lsr2 intergenic binding between convergent genes, neither gene was reported.

Table S5. Genes directly impacted by increased Lsr2 binding events in phosphoablative (T112A) Lsr2 *Mtb* compared to wild type Lsr2 *Mtb* using annotation from Mycobrowser (Release 3 2018-06-05).

A separate excel file

Genes were included in the predicted regulon if the Lsr2 binding site was within, or in the intergenic region upstream of, the coding sequence of each gene. For Lsr2 intergenic binding between divergent genes, both genes were reported

Table S6. NMR and refinement statistics for LSR2-WT and LSR2-T112D peptide structures (LSR2-WT and LSR2-T112D, 0.5 mM, 25 mM NaPhosphate pH 6.8, 150 mM NaCl, 293 K)

	**LSR2-WT**	**LSR2-T112D**
**NMR distance and dihedral constraints**		
Distance constraints		
Total NOE	616	482
Intra-residue	149	145
Inter-residue		
Sequential (|*i* – *j*| = 1)	163	127
Medium-range (|*i* – *j*| < 4)	158	102
Long-range (|*i* – *j*| > 5)	146	108
Hydrogen bonds	40	30
Total dihedral angle restraints		
ϕ	19	15
ψ	19	15
**Structure statistics**		
Violations (mean and s.d.)		
Max. distance constraint violation (Å)	0.18 ± 0.05	0.15 ± 0.03
Max. dihedral angle violation (º)	1.21 ± 1.46	0.45 ± 0.44
Deviations from idealized geometry		
Bond lengths (Å)	0.0098 ± 0.0005	0.0097 ± 0.0006
Bond angles (º)	1.1801 ± 0.0462	1.1952 ± 0.0663
Impropers (º)	1.2977 ± 0.1016	1.2639 ± 0.1166
**Ramachandran plot (%)**		
Most favoured region	87.8	80.6
Additionally allowed region	10.4	16.5
Generously allowed region	1.2	1.4
Disallowed region	0.6	1.4
**Average pairwise *r.m.s.* deviation** (Å)**		
Backbone	0.83 ± 0.31	1.13 ± 0.31
Heavy	1.72 ± 0.42	2.19 ± 0.46

** "Pairwise r.m.s. deviation calculated among 20 refined structures for residues 80-112."
